# Supplementary material for: The effects of genital myiasis on the diversity of the vaginal microbiota in female Bactrian camels
Source: BMC Vet Res. 2022 Mar 5;18:87. doi: 10.1186/s12917-022-03189-5 (PMC8897907; doi:10.1186/s12917-022-03189-5)
Supplement: Supplementary file 5 — Additional file 5. [file 12917_2022_3189_MOESM5_ESM.zip › MPL201709200_16s_yy/Treat1/css/js_css/jsPDF-master.zip!/jsPDF-master/examples/basic.html]

jsPDF


# jsPDF Demos

Examples for using jsPDF with Data URIs below. Go back to project homepage.

- Basic elements
- Text elements
- Graphic elements

## Simple two-page text document

```
var doc = new jsPDF();
doc.text(20, 20, 'Hello world!');
doc.text(20, 30, 'This is client-side Javascript, pumping out a PDF.');
doc.addPage();
doc.text(20, 20, 'Do you like that?');

doc.save('Test.pdf');
```

Run Code

## Landscape document

```
var doc = new jsPDF('landscape');
doc.text(20, 20, 'Hello landscape world!');

doc.save('Test.pdf');
```

Run Code

## Adding metadata

```
var doc = new jsPDF();
doc.text(20, 20, 'This PDF has a title, subject, author, keywords and a creator.');

// Optional - set properties on the document
doc.setProperties({
    title: 'Title',
    subject: 'This is the subject',
    author: 'James Hall',
    keywords: 'generated, javascript, web 2.0, ajax',
    creator: 'MEEE'
});

// Output as Data URI
doc.save('Test.pdf');
```

Run Code

## Example of user input

```
var name = prompt('What is your name?');
var multiplier = prompt('Enter a number:');
multiplier = parseInt(multiplier);

var doc = new jsPDF();
doc.setFontSize(22);
doc.text(20, 20, 'Questions');
doc.setFontSize(16);
doc.text(20, 30, 'This belongs to: ' + name);

for(var i = 1; i <= 12; i ++) {
    doc.text(20, 30 + (i * 10), i + ' x ' + multiplier + ' = ___');
}

doc.addPage();
doc.setFontSize(22);
doc.text(20, 20, 'Answers');
doc.setFontSize(16);

for(var i = 1; i <= 12; i ++) {
    doc.text(20, 30 + (i * 10), i + ' x ' + multiplier + ' = ' + (i * multiplier));
}
doc.save('Test.pdf');
```

Run Code

## Different font sizes

```
var doc = new jsPDF();
doc.setFontSize(22);
doc.text(20, 20, 'This is a title');

doc.setFontSize(16);
doc.text(20, 30, 'This is some normal sized text underneath.');

doc.save('Test.pdf');
```

Run Code

## Different font types

```
var doc = new jsPDF();

doc.text(20, 20, 'This is the default font.');

doc.setFont("courier");
doc.text(20, 30, 'This is courier normal.');

doc.setFont("times");
doc.setFontType("italic");
doc.text(20, 40, 'This is times italic.');

doc.setFont("helvetica");
doc.setFontType("bold");
doc.text(20, 50, 'This is helvetica bold.');

doc.setFont("courier");
doc.setFontType("bolditalic");
doc.text(20, 60, 'This is courier bolditalic.');

doc.save('Test.pdf');
```

Run Code

## Different text colors

```
var doc = new jsPDF();

doc.setTextColor(100);
doc.text(20, 20, 'This is gray.');

doc.setTextColor(150);
doc.text(20, 30, 'This is light gray.');

doc.setTextColor(255,0,0);
doc.text(20, 40, 'This is red.');

doc.setTextColor(0,255,0);
doc.text(20, 50, 'This is green.');

doc.setTextColor(0,0,255);
doc.text(20, 60, 'This is blue.');

doc.save('Test.pdf');
```

Run Code

## Font-metrics-based line sizing and split

```
var pdf = new jsPDF('p','in','letter')
, sizes = [12, 16, 20]
, fonts = [['Times','Roman'],['Helvetica',''], ['Times','Italic']]
, font, size, lines
, verticalOffset = 0.5 // inches on a 8.5 x 11 inch sheet.
, loremipsum = 'Lorem ipsum dolor sit amet, ...'

for (var i in fonts){
    if (fonts.hasOwnProperty(i)) {
        font = fonts[i]
        size = sizes[i]

        lines = pdf.setFont(font[0], font[1])
                    .setFontSize(size)
                    .splitTextToSize(loremipsum, 7.5)
        // Don't want to preset font, size to calculate the lines?
        // .splitTextToSize(text, maxsize, options)
        // allows you to pass an object with any of the following:
        // {
        //  'fontSize': 12
        //  , 'fontStyle': 'Italic'
        //  , 'fontName': 'Times'
        // }
        // Without these, .splitTextToSize will use current / default
        // font Family, Style, Size.

        pdf.text(0.5, verticalOffset + size / 72, lines)

        verticalOffset += (lines.length + 0.5) * size / 72
    }
}

pdf.save('Test.pdf');
```

Run Code

## fromHTML plugin

This (BETA level. API is subject to change!) plugin allows one to scrape formatted text from an HTML fragment into PDF. Font size, styles are copied. The long-running text is split to stated content width.

## Header Two

***Double style span***
Monotype span with
carriage return. a humongous font size span.
Followed by long parent-less text node. asdf qwer asdf zxcv qsasfd qwer qwasfd zcxv sdf qwer qwe sdf wer qwer asdf zxv.

Serif Inner DIV (bad markup, but testing block detection)

 Sans-serif span with extra spaces 
Followed by text node without any wrapping element. And some long long text span attached at the end to test line wrap. qwer asdf qwer lkjh asdf zxvc safd qwer wwwwwwwwwwwwwwwwwwwwwwwwwwwwwwwwwwwwwwwwwwwwwwwwwwwwwwwwwwwwwwwwwwwwwwwwwwwwwwwwwwwwwwwwwwwwwwwwwwwwwwwwwwwwwwwwwwwwwwwwwwwwwwwwwwwwwwwwwwwwwwwwwwwwwwwwwwwwwwwwwwwwwwwww qewr asdf zxcv.

This is a *new* paragraph.

This is more wrapping-less text.

This paragraph will **NOT** be on resulting PDF because a special attached element handler will be looking for the ID - 'bypassme' - and should bypass rendering it.

This is **another** paragraph.

Integer dignissim urna tortor? Cum rhoncus, a lacus ultricies tincidunt, tristique lundium enim urna, magna? Sed, enim penatibus? Lacus pellentesque integer et pulvinar tortor? Dapibus in arcu arcu, vut dolor? Et! Placerat pulvinar cursus, urna ultrices arcu nunc, a ultrices dictumst elementum? Magnis rhoncus pellentesque, egestas enim purus, augue et nascetur sociis enim rhoncus. Adipiscing augue placerat tincidunt pulvinar ridiculus. Porta in sociis arcu et placerat augue sit enim nec hac massa, turpis ridiculus nunc phasellus pulvinar proin sit pulvinar, ultrices aliquet placerat amet? Lorem nunc porttitor etiam risus tempor placerat amet non hac, nunc sed odio augue? Turpis, magnis. Lorem pid, a porttitor tincidunt adipiscing sagittis pellentesque, mattis amet, duis proin, penatibus lectus lorem eros, nisi, tempor phasellus, elit.

## Image Support

NOTES: the img src must be on the same domain or the external domain should allow Cross-origin.


## New page added with html comment: ADD\_PAGE

HTML Table:

NOTES: Must set the COLGROUP tag with "with" on each COL tag as %, inspect the table. BTW the css does not have a good style to render the table on the html :P, feel free to the add the CSS.

| Heading1 | Heading2 |
| --- | --- |
| cell 1,1 | cell 1,2 |
| cell 2,1 | cell 2,2 |
| cell 3,1 | cell 3,2 |
| cell 4,1 | cell 4,2 |

HTML Lists:

- Lorem Ipsum
- Dolor Sit amen
- Lorem Ipsum
- Dolor Sit amen

1. Lorem Ipsum
2. Dolor Sit amen
3. Lorem Ipsum
4. Dolor Sit amen

```
var pdf = new jsPDF('p', 'pt', 'letter')

// source can be HTML-formatted string, or a reference
// to an actual DOM element from which the text will be scraped.
, source = $('#fromHTMLtestdiv')[0]

// we support special element handlers. Register them with jQuery-style
// ID selector for either ID or node name. ("#iAmID", "div", "span" etc.)
// There is no support for any other type of selectors
// (class, of compound) at this time.
, specialElementHandlers = {
    // element with id of "bypass" - jQuery style selector
    '#bypassme': function(element, renderer){
        // true = "handled elsewhere, bypass text extraction"
        return true
    }
}

margins = {
    top: 80,
    bottom: 60,
    left: 40,
    width: 522
  };
  // all coords and widths are in jsPDF instance's declared units
  // 'inches' in this case
pdf.fromHTML(
    source // HTML string or DOM elem ref.
    , margins.left // x coord
    , margins.top // y coord
    , {
        'width': margins.width // max width of content on PDF
        , 'elementHandlers': specialElementHandlers
    },
    function (dispose) {
      // dispose: object with X, Y of the last line add to the PDF
      //          this allow the insertion of new lines after html
        pdf.save('Test.pdf');
      },
    margins
  )
```

Run Code

## Text alignment

```
var pdf = new jsPDF('p', 'pt', 'letter');
    
    pdf.text( 'This text is normally\raligned.', 140, 50 );
    
    pdf.text( 'This text is centered\raround\rthis point.', 140, 120, 'center' );
    
    pdf.text( 'This text is rotated\rand centered around\rthis point.', 140, 300, 45, 'center' );
    
    pdf.text( 'This text is\raligned to the\rright.', 140, 400, 'right' );
    
    pdf.text( 'This text is\raligned to the\rright.', 140, 550, 45, 'right' );
    
    pdf.text( 'This single line is centered', 460, 50, 'center' );

    pdf.text( 'This right aligned text\r\rhas an empty line.', 460, 200, 'right' );
    
    
    pdf.save('Test.pdf');
```

Run Code

## Draw example: rectangles / squares

```
var doc = new jsPDF();

doc.rect(20, 20, 10, 10); // empty square

doc.rect(40, 20, 10, 10, 'F'); // filled square

doc.setDrawColor(255,0,0);
doc.rect(60, 20, 10, 10); // empty red square

doc.setDrawColor(255,0,0);
doc.rect(80, 20, 10, 10, 'FD'); // filled square with red borders

doc.setDrawColor(0);
doc.setFillColor(255,0,0);
doc.rect(100, 20, 10, 10, 'F'); // filled red square

doc.setDrawColor(0);
doc.setFillColor(255,0,0);
doc.rect(120, 20, 10, 10, 'FD'); // filled red square with black borders

doc.setDrawColor(0);
doc.setFillColor(255, 255, 255);
doc.roundedRect(140, 20, 10, 10, 3, 3, 'FD'); //  Black square with rounded corners

doc.save('Test.pdf');
```

Run Code

## Draw example: lines

```
var doc = new jsPDF();

doc.line(20, 20, 60, 20); // horizontal line

doc.setLineWidth(0.5);
doc.line(20, 25, 60, 25);

doc.setLineWidth(1);
doc.line(20, 30, 60, 30);

doc.setLineWidth(1.5);
doc.line(20, 35, 60, 35);

doc.setDrawColor(255,0,0); // draw red lines

doc.setLineWidth(0.1);
doc.line(100, 20, 100, 60); // vertical line

doc.setLineWidth(0.5);
doc.line(105, 20, 105, 60);

doc.setLineWidth(1);
doc.line(110, 20, 110, 60);

doc.setLineWidth(1.5);
doc.line(115, 20, 115, 60);

doc.save('Test.pdf');
```

Run Code

## Draw example: circles and ellipses

```
var doc = new jsPDF();

doc.ellipse(40, 20, 10, 5);

doc.setFillColor(0,0,255);
doc.ellipse(80, 20, 10, 5, 'F');

doc.setLineWidth(1);
doc.setDrawColor(0);
doc.setFillColor(255,0,0);
doc.circle(120, 20, 5, 'FD');

doc.save('Test.pdf');
```

Run Code

## Draw example: triangles

```
var doc = new jsPDF();

doc.triangle(60, 100, 60, 120, 80, 110, 'FD');

doc.setLineWidth(1);
doc.setDrawColor(255,0,0);
doc.setFillColor(0,0,255);
doc.triangle(100, 100, 110, 100, 120, 130, 'FD');

doc.save('My file.pdf');
```

Run Code

## Draw example: Images

```
// Because of security restrictions, getImageFromUrl will
// not load images from other domains.  Chrome has added
// security restrictions that prevent it from loading images
// when running local files.  Run with: chromium --allow-file-access-from-files --allow-file-access
// to temporarily get around this issue.
var getImageFromUrl = function(url, callback) {
    var img = new Image();

    img.onError = function() {
        alert('Cannot load image: "'+url+'"');
    };
    img.onload = function() {
        callback(img);
    };
    img.src = url;
}

// Since images are loaded asyncronously, we must wait to create
// the pdf until we actually have the image.
// If we already had the jpeg image binary data loaded into
// a string, we create the pdf without delay.
var createPDF = function(imgData) {
    var doc = new jsPDF();

    // This is a modified addImage example which requires jsPDF 1.0+
    // You can check the former one at examples/js/basic.js

    doc.addImage(imgData, 'JPEG', 10, 10, 50, 50, 'monkey'); // Cache the image using the alias 'monkey'
    doc.addImage('monkey', 70, 10, 100, 120); // use the cached 'monkey' image, JPEG is optional regardless
    // As you can guess, using the cached image reduces the generated PDF size by 50%!

    // Rotate Image - new feature as of 2014-09-20
    doc.addImage({
        imageData : imgData,
        angle     : -20,
        x         : 10,
        y         : 78,
        w         : 45,
        h         : 58
    });

    // Output as Data URI
    doc.output('datauri');
}

getImageFromUrl('thinking-monkey.jpg', createPDF);
```


Run Code
